# Capturing respiratory syncytial virus season in Belgium using the influenza severe acute respiratory infection surveillance network, season 2018/19

**DOI:** 10.2807/1560-7917.ES.2020.25.39.1900627

**Published:** 2020-10-01

**Authors:** Lorenzo Subissi, Nathalie Bossuyt, Marijke Reynders, Michèle Gérard, Nicolas Dauby, Marc Bourgeois, Bénédicte Delaere, Sophie Quoilin, Steven Van Gucht, Isabelle Thomas, Cyril Barbezange

**Affiliations:** 1 National Influenza Centre, Sciensano, Brussels, Belgium; 2 European Public Health Microbiology Training Programme (EUPHEM), European Centre for Disease Prevention and Control, Stockholm, Sweden; 3 Epidemiology of Infectious Diseases, Sciensano, Brussels, Belgium; 4 Department of Laboratory Medicine, Medical Microbiology, Algemeen Ziekenhuis Sint-Jan, Brugge-Oostende AV, Belgium; 5 Centre Hospitalier Universitaire St-Pierre, Brussels, Belgium; 6 Centre for Environmental Health and Occupational Health, School of Public Health, Université Libre de Bruxelles (ULB), Brussels, Belgium; 7 Centre Hospitalier Universitaire UCL Namur, Ysoir, Belgium

**Keywords:** respiratory syncytial virus, surveillance, severe acute respiratory infection, influenza virus

## Abstract

**Background:**

Respiratory syncytial virus (RSV) is a common cause of severe respiratory illness in young children (< 5 years old) and older adults (≥ 65 years old) leading the World Health Organization (WHO) to recommend the implementation of a dedicated surveillance in countries.

**Aim:**

We tested the capacity of the severe acute respiratory infection (SARI) hospital network to contribute to RSV surveillance in Belgium.

**Methods:**

During the 2018/19 influenza season, we started the SARI surveillance for influenza in Belgium in week 40, earlier than in the past, to follow RSV activity, which usually precedes influenza virus circulation. While the WHO SARI case definition for influenza normally used by the SARI hospital network was employed, flexibility over the fever criterion was allowed, so patients without fever but meeting the other case definition criteria could be included in the surveillance.

**Results:**

Between weeks 40 2018 and 2 2019, we received 508 samples from SARI patients. We found an overall RSV detection rate of 62.4% (317/508), with rates varying depending on the age group: 77.6% in children aged < 5 years (253/326) and 34.4% in adults aged ≥ 65 years (44/128). Over 90% of the RSV-positive samples also positive for another tested respiratory virus (80/85) were from children aged < 5 years. Differences were also noted between age groups for symptoms, comorbidities and complications.

**Conclusion:**

With only marginal modifications in the case definition and the period of surveillance, the Belgian SARI network would be able to substantially contribute to RSV surveillance and burden evaluation in children and older adults, the two groups of particular interest for WHO.

## Introduction

Respiratory syncytial virus (RSV) is an important cause of acute lower respiratory infections in children [1]. Severe forms of disease caused by RSV, including pneumonia, can lead to hospitalisation resulting in several thousand deaths per year worldwide in hospitalised children under the age of 5 years [2]. RSV is also a major cause of severe acute respiratory infections (SARI) in older adults (≥ 65 years old) [3-5]. Currently, in Europe, palivizumab is the only commercially-available antiviral designed against RSV infection, and specifically for children [6]. Vaccines are in development and are expected to become available in the coming years [7-9]. To help decision-makers and to evaluate the impact of future vaccination, a dedicated surveillance is necessary. This surveillance might shed light on several aspects of RSV epidemiology (e.g. seasonality, at-risk age groups, complications) in particular why/how certain parameters vary between different areas of the world [10].

In 2016, the World Health Organization (WHO) launched a pilot study to evaluate the feasibility of using the long-standing worldwide influenza surveillance network (Global Influenza Surveillance and Response System) for a sustainable RSV surveillance [10]. The attempt to implement such a surveillance aimed at better understanding the virus, its circulation patterns and the disease it causes in the different age groups to eventually refine case definitions [10]. In addition, this study tried to look into the health burden of RSV in order to identify at-risk groups who would most benefit from vaccination [10]. The case definition recommended for countries using a hospital-based surveillance strategy was an extended WHO SARI case definition for patients aged ≥ 6 months. For those aged < 6 months, the case definition should also include apnoea or sepsis [10]. Moreover, the sampling strategy recommended ca 1,000 samples per year per country, distributed among the following age groups: < 6 months, 6 months to 4 years, 5 to 64 years, and ≥ 65 years [10].

In Belgium, the national influenza centre recognised by WHO obtains influenza surveillance data through two networks. The first consists of general practitioners and concerns influenza-like illness (ILI) surveillance (from week 40 to week 20 of the following year; i.e. influenza season surveillance period); the second is constituted by six hospitals that monitor SARI (from the last week of December or first/second week of January to the third/last week of April, depending on when influenza virus circulation is detected by the ILI network; i.e. the influenza activity period). Historical data of the numbers of positive tests reported by all the Belgian hospital laboratories show that RSV circulation generally occurs sometime between October and January, and usually precedes or slightly overlaps the circulation of influenza viruses [11], like in neighbouring countries [12,13].

Since these data do not provide enough information to assess the RSV burden, we decided to test the existing SARI network capability to contribute to RSV surveillance in Belgium.

## Methods

### Settings of the Belgian pilot study

During the 2018/19 influenza season (week 40 2018 to week 20 2019), three of the six hospitals of the SARI network, one in each administrative region (Flanders, Brussels-Capital, Wallonia), volunteered to start the SARI surveillance as early as week 40 2018, instead of at the end of December or beginning of January. Our usual SARI case definition, based on the WHO SARI case definition, was used: acute respiratory infection with fever ≥ 38 °C (or history of fever reported by the patient) and cough or dyspnoea, with onset of symptoms within the past 10 days, and requiring hospitalisation (minimum overnight). However, in order to align with one of the case definitions proposed in WHO’s RSV strategy document [14,15], the participating hospitals were recommended to be flexible over the fever criteria, meaning that patients without fever but meeting the other criteria could also be included.

Unless consent was refused, enrolment included all patients meeting the case definition. Beside sampling (nasopharyngeal swab or aspirate), SARI standardised questionnaires were used to collect data on age, sex, symptoms, antibiotic treatment, known comorbidities and follow-up during hospitalisation to evaluate the disease severity. 

### Laboratory investigation

Respiratory samples were analysed at the national influenza centre. Viral nucleic acids were extracted using BioMerieux’s NucliSENS EasyMag (Brussels, Belgium). Routine in-house multiplex reverse transcription quantitative (RT-q)PCRs were used to detect the following respiratory virus targets: adenoviruses, bocavirus, coronaviruses (CoV-OC43, CoV-NL63 and CoV-229E separately), human metapneumoviruses, influenza virus types A and B, parainfluenzavirus types 1, 2, 3 and 4 (separately), parechovirus, picornaviruses (rhinovirus and enterovirus genera) with specifically enterovirus D68, and RSV types A and B (adapted from original protocols by United States Centers for Disease Control and Prevention; LJ van Elden, University Medical Centre Utrecht, the Netherlands; P Overduin, the National Institute for Public Health and the Environment, the Netherlands; O Hungnes and K Bragstad, Institute of Public Health, Norway). Primer and probe sequences and RT-qPCR conditions are available upon request.

### Statistical analysis

A two-tailed Fisher’s exact test (two-by-two table) or a chi-squared test was performed for group proportion comparisons. The Mann–Whitney test was used to compare the distributions of the length of stay in hospital. Differences were considered as statistically significant for a p value < 0.05.

### Ethical statement

The SARI surveillance protocol was approved by the central Ethical Committee (reference AK/12–02–11/4111; in 2011: Centre Hospitalier Universitaire St-Pierre, Brussels, Belgium; since 2014: Universitair Ziekenhuis Brussel, Brussels, Belgium) and the local ethical committees of the hospitals. The amendment for the pilot study was specifically approved by the central Ethical Committee and the local ethical committees of the participating hospitals. Informed consent was obtained from all participants or parents/guardians.

## Results

### Capturing the respiratory syncytial virus season through the SARI network

**Figure 1:**
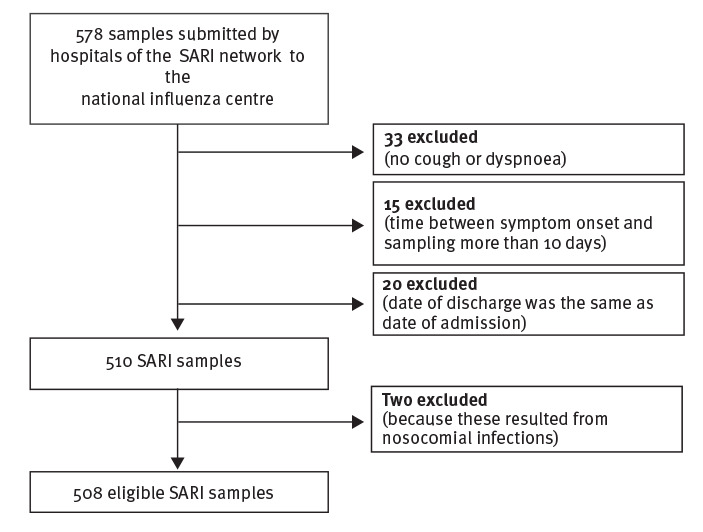
Flowchart for the inclusion of SARI patients in the RSV pilot study, Belgium, October 2018–January 2019 (n = 508 included patients)^a^

RSV: respiratory syncytial virus; SARI: severe acute respiratory infection.

^a^ Only three of the six hospitals in the Belgian SARI surveillance network, one in each administrative region (Flanders, Brussels-Capital, Wallonia), took part in this pilot study.

From week 40 2018 until week 2 2019, the week of the official start of the influenza SARI surveillance for the six hospitals, the national influenza centre received a total of 578 samples from the three hospitals participating in the study, of which 508 were eligible based on the adopted case definition (Figure 1). The median number of samples per week was 38 (interquartile range (IQR): 18.5–37.8). The three sites each contributed to 36.0% (183/508), 39.2% (199/508) and 24.8% (126/508) of the total samples. There were slightly more samples from males (55.1%, 280/508) than females. The WHO’s recommended four age groups were covered, but the patients aged between 5 and 64 years were less represented (Table 1) and differences between sites were noted (data not shown).

**Table 1:** Characteristics of SARI patients according to RSV and other respiratory virus test results, Belgium, week 41 2018–week 2 2019 (n = 508)

Characteristic		RSV negative				RSV positive				Total	
		Negative for all other respiratory virus tested		Positive for another respiratory virus tested^a^		RSV only		RSV co-infection^a^			
		Number	%	Number	%	Number	%	Number	%	Number	%
Overall		82	100	109	100	232	100	85	100	508	100
Age group	< 6 m	6	7.3	24	22.0	117	50.4	42	49.4	189	37.2
	6 m–4 y	12	14.6	31	28.4	56	24.1	38	44.7	137	27.0
	5–64 y	22	26.8	12	11.0	17	7.3	3	3.5	54	10.6
	≥ 65 y	42	51.2	42	38.5	42	18.1	2	2.4	128	25.0
Symptom	Fever	65	79.3	96	88.1	211	90.9	82	96.5	454	89.4
	Cough	66	80.5	96	88.1	213	91.8	75	88.2	450	88.6
	Dyspnoea	60	73.2	66	60.6	126	54.3	44	51.8	296	58.3
Comorbidity	No	20	24.4	45	41.3	143	61.6	65	76.5	273	53.7
	Yes	62	75.6	64	58.7	89	38.4	20	23.5	83	16.3
	Chr. respi.	27	32.9	22	20.2	28	12.1	6	7.1	83	16.3
	Asthma	5	6.1	7	6.4	10	4.3	3	3.5	25	4.9
	Chr. cardio.	18	22.0	24	22.0	21	9.1	2	2.4	65	12.8
	Renal insuf.	11	13.4	13	11.9	15	6.5	3	3.5	42	8.3
	Hep. insuf.	4	4.9	6	5.5	4	1.7	0	0.0	14	2.8
	Obesity	13	15.9	5	4.6	4	1.7	1	1.2	23	4.5
	Diabetes	8	9.8	12	11.0	8	3.5	2	2.4	30	5.9
	Immunodef.	6	7.3	15	13.8	22	9.5	3	3.5	46	9.1
	Neuromusc.	5	6.1	10	9.2	12	5.2	0	0.0	27	5.3
	Unknown	2	ND	0	ND	0	ND	0	ND	2	ND
Antibiotics	No	26	31.7	36	33.0	120	51.7	55	64.7	237	46.7
	Yes	53	64.6	69	63.3	103	44.4	30	35.3	255	50.2
	Unknown	1	1.2	0	0.0	1	0.4	0	0.0	2	0.4
	Missing	2	ND	4	ND	8	ND	0	ND	14	ND
Death	All ages	7	8.5	8	7.3	6	2.6	1	1.2	22	4.3
	5–64 y	1	ND	2	ND	0	ND	1	ND	4	ND
	≥ 65 y	6	ND	6	ND	6	ND	0	ND	18	ND
Complication^b^	No	45	54.9	62	56.9	126	54.3	50	58.8	283	55.7
	Yes^c^	37	45.1	47	43.1	106	45.7	35	41.2	225	44.3
	Pneumonia	19	ND	26	ND	36	ND	11	ND	92	ND
	ICU	15	ND	11	ND	21	ND	4	ND	51	ND
	ARDS	5	ND	10	ND	14	ND	5	ND	34	ND
	Resp. assis.	19	ND	24	ND	77	ND	25	ND	145	ND
Stay in hospital^d^	Median	6	NA	4	NA	5	NA	4	NA	6	NA
	Min	1	NA	1	NA	1	NA	1	NA	1	NA
	25% perc.	3.8	NA	3	NA	3	NA	3	NA	3	NA
	75% perc.	12	NA	10.5	NA	8	NA	6	NA	8	NA
	Max	37	NA	104	NA	56	NA	14	NA	104	NA

ARDS: acute respiratory distress syndrome; chr. cardio.: chronic cardiovascular disease; chr. respi.: chronic respiratory disease; hep. insuf.: hepatic insufficiency; ICU: intensive care unit; immunodef.: immunodeficiency; m: months; max: maximum; min: minimum; NA: not applicable; ND: not determined; neuromusc.: neuromuscular disease; perc.: percentile; renal insuf.: renal insufficiency; resp. assis.: requirement for respiratory assistance (invasive and non-invasive); RSV: respiratory syncytial virus; SARI: severe acute respiratory infection; y: years.

^a^ Other respiratory viruses tested: adenoviruses, bocavirus, coronaviruses (CoV-OC43, CoV-NL63 and CoV-229E), human metapneumoviruses, influenza virus types A and B, parainfluenzaviruses (types 1, 2, 3 and 4), parechovirus, picornaviruses of the rhinovirus and enterovirus genera, specific enterovirus D68.

^b^ Other than death.

^c^ At least one of the following: admission to ICU, detection of pneumonia based on chest radiography, development of ARDS, requirement for respiratory assistance.

^d^ In days.

Number of patients and percentage within laboratory result category are presented.

**Figure 2:**
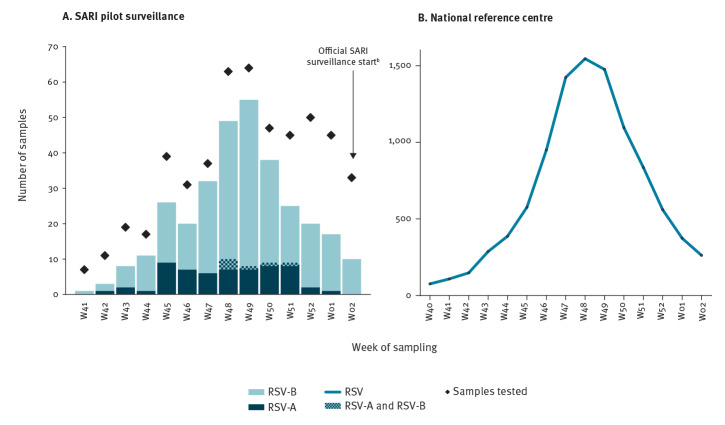
Weekly number of samples testing positive for RSV (A) captured by the SARI pilot surveillance and (B) reported by the NRC^a^, Belgium, week 41 2018–week 2 2019 (n = 508)

NRC: national reference centre for respiratory pathogens; RSV: respiratory syncytial virus; SARI: severe acute respiratory infection.

^a^ The distribution is based on the total number of RSV-positive tests reported to the NRC by the hospital laboratories in Belgium (including outpatients and inpatients, without any case definition; typing was not performed).

^b^ The arrow indicates the start of the seasonal official SARI surveillance, which, in 2019, began in week 2. Compared to the pilot surveillance, the official SARI surveillance involves three additional hospitals.

The weekly distribution of the number of RSV-positive samples among the SARI patients (Figure 2A) appeared to match the epidemiological curve reported by the national reference centre for respiratory pathogens (Figure 2B). Among the SARI patients, the peak of RSV infections was reached at week 49 with 55 RSV-positive samples (of 64 tested). Overall, during the study period, 62.4% (317/508) of the samples were positive for RSV, with RSV-B dominating during the 2018/19 season. 

For the SARI surveillance (panel A), the weekly number of samples tested are represented by diamonds. The number of RSV-positive samples in the histograms are stacked, with different colours representing samples positive for RSV-A, RSV-B, or both RSV-A and RSV-B.

**Figure 3:**
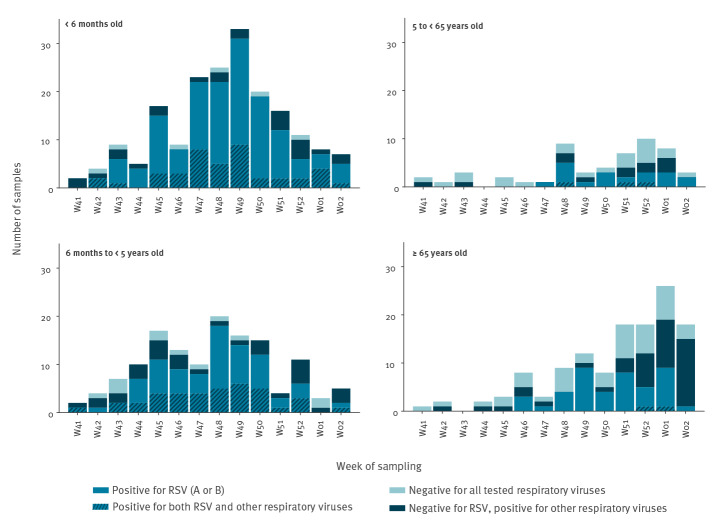
Weekly RSV detection among SARI patients by age group, Belgium, week 41 2018–week 2 2019

SARI: severe acute respiratory infection; RSV: respiratory syncytial virus; WHO: World Health Organization.

Detection rates decreased with age (Table 1): 84.1% among infants aged < 6 months (159/189), 68.6% among the 6-month-to-4-year-old children (94/137), 37.0% among the 5-to-64-year-old patients (20/54) and 34.4% among the older adults (44/128). The weekly distributions of the number of positive samples were similar for the two younger age groups, with a clear bell-shape curve starting as early as week 41 (Figure 3). On the other hand, for the 5-to-64- and ≥ 65-year age groups, the first positive samples were detected only from week 47 and 46, respectively, and the curve resulting from the distribution did not present a clear peak.

Age groups were defined as in the WHO’s pilot study documents. In the histograms, numbers of samples per result categories, represented in different colours, are stacked (cumulative).

Among the RSV-positive samples (Table 1), 26.8% (85/317) were also positive for at least one other respiratory virus tested, while this proportion was 57.1% among the RSV-negative samples (109/191). There was very good evidence of differences between age groups in the proportion of positivity (Table 1; chi-squared test p < 0.001). Among children aged < 5 years, 53.1% (173/326) tested positive only for RSV, 24.5% (80/326) tested positive for RSV and at least one other respiratory virus investigated, nearly 17% (55/326) tested positive for a respiratory virus other than RSV (Supplement Table S1), and less than 6% (18/326) were negative for all tested respiratory viruses. In contrast, among patients aged ≥ 5 years, more than one third (64/182) tested negative for all investigated respiratory viruses, 32% (59/182) tested positive for only RSV, nearly 30% (54/182) tested positive only for a respiratory virus other than RSV, and less than 3% (5/182) tested positive for RSV and at least one other respiratory virus of the panel (Supplement Table S1).

### Clinical signs included in the SARI case definition and other symptoms

**Figure 4:**
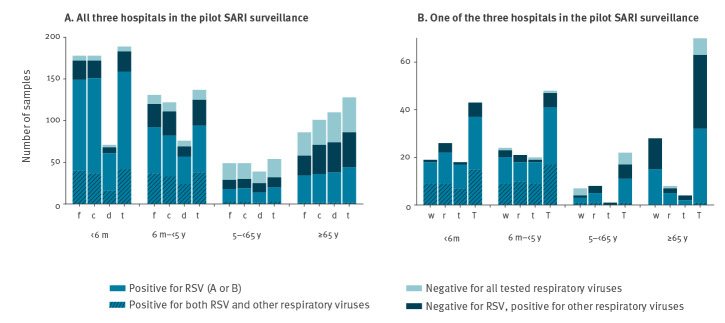
Clinical signs among SARI patients per age group, according to data from (A) all three hospitals^a^ participating in the study and (B) one of these three hospitals^b^, Belgium, week 41 2018–week 2 2019

The data for fever, cough and dyspnoea are presented in Table 2 and Supplement Table S1, and summarised in Figure 4A. Fever was more common among children aged < 5 years than among patients aged ≥ 5 years regardless of the RSV status (with fever, RSV-negative: 93.2% (68/73) for < 5 years old vs 78.8% (93/118) for ≥ 5 years old, Fisher exact test p = 0.008; with fever, RSV-positive: 95.3% (241/253) for < 5 years old vs 81.3% (52/64) for ≥ 5 years old, Fisher exact test p < 0.001) (Table 2). No significant difference was observed in the proportions with fever among RSV-negative and RSV-positive patients, whatever the age group. There was some evidence of a difference in the proportion of patients with cough between the RSV-positive group (90.9%, 288/317) and the RSV-negative group (84.8%, 162/191, Fisher exact test p = 0.044) (Table 2). However, this difference was not observed when comparing within each age group. There was very good evidence that dyspnoea was more often reported for patients aged ≥ 5 years (81.9% (149/182)) than among patients aged < 5 years (45.1% (147/326)), Fisher exact test p < 0.001, but without difference between RSV-positive and RSV-negative cases (Table 2).

**Table 2:** Characteristics of SARI patients according to age group and by result category, Belgium, week 41 2018–week 2 2019 (n = 508)

Characteristic			RSV negative		RSV positive		Total	
			Number	%	Number	%	Number	%
Overall			191	100	317	100	508	100
Age	< 5 y		73	38.2	253	79.8	326	64.2
	≥ 5 y		118	61.8	64	20.2	182	35.8
Symptoms	All ages	Fever	161	84.3	293	92.4	454	89.4
		Cough	162	84.8	288	90.9	450	88.6
		Dyspnoea	126	66.0	170	53.6	296	58.3
	< 5 y	Fever	68	93.2	241	95.3	309	94.8
		Cough	67	91.8	233	92.1	300	92.0
		Dyspnoea	29	39.7	118	46.6	147	45.1
	≥ 5 y	Fever	93	78.8	52	81.3	145	79.7
		Cough	95	80.5	55	85.9	150	82.4
		Dyspnoea	97	82.2	52	81.3	149	81.9
Comorbidity^a^	All ages	None	65	34.0	208	65.6	273	53.7
		Yes	126	66.0	109	34.4	83	16.3
	< 5 y	None	56	76.7	205	81.0	261	80.1
		Yes	17	23.3	48	19.0	65	19.9
	≥ 5 y	None	9	7.6	4	6.3	13	7.1
		Yes	109	92.4	60	93.8	169	92.9
Complications^b^	All ages	No	107	56.0	176	55.5	283	55.7
		Yes^c^	84	44.0	141	44.5	225	44.3
		Pneumonia	45	ND	47	ND	92	ND
		ICU	26	ND	25	ND	51	ND
		ARDS	15	ND	19	ND	34	ND
		Resp. assis.	43	ND	102	ND	145	ND
	< 5 y	No	46	63.0	152	60.1	198	60.7
		Yes^c^	27	37.0	101	39.9	128	39.3
		Pneumonia	8	ND	25	ND	33	ND
		ICU	0	ND	6	ND	6	ND
		ARDS	3	ND	12	ND	15	ND
		Resp. assis.	21	ND	82	ND	103	ND
	≥ 5 y	No	61	51.7	24	37.5	85	46.7
		Yes^c^	57	48.3	40	62.5	97	53.3
		Pneumonia	37	ND	22	ND	59	ND
		ICU	26	ND	19	ND	45	ND
		ARDS	12	ND	7	ND	19	ND
		Resp. assis.	22	ND	20	ND	42	ND
Death	All ages		15	7.9	7	2.2	22	4.3
	5–64 y		3	8.8	1	5.0	4	7.4
	≥ 65 y		12	14.3	6	13.6	18	14.1
Length of stay^d^	All ages	Median	5	NA	5	NA	5	NA
		Min	1	NA	1	NA	1	NA
		25% percentile	3	NA	3	NA	3	NA
		75% percentile	11	NA	7	NA	8	NA
		Max	104	NA	56	NA	104	NA
	< 5 y	Median	3	NA	4	NA	4	NA
		Min	1	NA	1	NA	1	NA
		25% percentile	2	NA	3	NA	2	NA
		75% percentile	4	NA	6	NA	5	NA
		Max	19	NA	32	NA	32	NA
	≥ 5 y	Median	9	NA	8.5	NA	9	NA
		Min	1	NA	1	NA	1	NA
		25% percentile	5	NA	5	NA	5	NA
		75% percentile	16	NA	14.8	NA	15.3	NA
		Max	104	NA	56	NA	104	NA

ARDS: acute respiratory distress syndrome; ICU: intensive care unit; max: maximum; min: minimum; NA: non applicable; ND: not determined; Resp. assis.: requirement for respiratory assistance (invasive and non-invasive); RSV: respiratory syncytial virus; SARI: severe acute respiratory infection; y: year.

^a^ At least one of the following: asthma, chronic cardiovascular disease, chronic respiratory disease, diabetes, exposure to tobacco, hepatic insufficiency, immunodeficiency, neuromuscular disease, obesity, prematurity, renal insufficiency.

^b^ Other than death.

^c^ At least one of the following: admission in ICU, detection of pneumonia based on chest radiography, development of ARDS, resp. assis..

^d^ In days.

C: cough; d: dyspnoea; f: fever; m: month; SARI: severe acute respiratory infection; r: rhinitis; t: tachypnoea; T: total; w: wheezing; WHO: World Health Organization; y: year.

^a^ All three hospitals reported for each SARI case the clinical signs that were present and included in the WHO SARI case definition (cough or dyspnoea and/or fever).

^b^ One hospital reported, for all its patients, some additional clinical signs to those in the WHO SARI case definition. These included rhinitis, tachypnoea and wheezing, as shown in panel B.

Age groups were defined as in the WHO’s pilot study documents. In the histograms, numbers of samples per result categories, represented in different colours, are stacked (cumulative).

In addition to these clinical signs included in the case definition and stated on the questionnaire, hospital site 1 systematically reported for all patients a detailed list of other symptoms registered by the medical staff in the patients’ files. Some of these additional symptoms have been positively associated to RSV in another study [16]. Figure 4B summarises the results of hospital site 1 for rhinitis, tachypnoea and wheezing. These three symptoms appeared to be more frequently reported for RSV-positive patients in the < 5-year-olds (respectively: 48.7% (38/78), 44.9% (35/78), 42.3% (33/78) for RSV-positive vs 23.1% (3/13), 23.1% (3/13), 15.4% (2/13) for RSV-negative). In the ≥ 5-year-olds, differences between RSV-positive and RSV-negative were not as clear (respectively: 41.9% (18/43), 16.3% (7/43), 4.6% (2/43) for RSV-positive vs 34.7% (17/49), 4.1% (2/49), 6.1% (3/49) for RSV-negative).

### Risk factors and complications during hospitalisation

Comorbidities were more common in patients aged ≥ 5 years than in those aged < 5 years (92.9% (169/182) vs 19.9% (65/326), two-sided Fisher exact test p < 0.001), but no difference was observed between RSV-positive and RSV-negative patients within each age group (Table 2). Supplement Table S1 details the comorbidities per age group. Among the comorbidities reported in children < 6 months (24/189), the most common were premature birth (nine patients, six of whom RSV-positive), chronic respiratory diseases (five patients, all RSV-positive), tobacco exposure (four patients, three of whom RSV-positive) and chronic cardiovascular disease (one RSV-positive patient). Among the most reported comorbidities in the 6-month-to-4-year-old patients (42/137), nine were classified as premature birth (seven RSV-positive), seven as chronic respiratory diseases (five RSV-positive), six as asthma (four RSV-positive), six as neuromuscular diseases (five RSV-positive), five as immunodeficiencies (four RSV-positive), three as tobacco exposure (all RSV-positive) and two as chronic cardiovascular diseases (both RSV-positive). The most frequently reported comorbidities in patients aged ≥ 5 years were chronic respiratory diseases (71/169), chronic cardiovascular diseases (62/169), immunodeficiency (41/169), diabetes (30/169), obesity (23/169) and asthma (19/169). Cases were relatively evenly distributed between RSV-positive, negative for all tested viruses, and only positive for another tested respiratory virus (Supplement Table S1).

There was very good evidence that the hospitalisation stay of patients aged ≥ 5 years (median: 9 days; IQR: 5–15) was longer than that of the patients aged < 5 years (median: 4 days; IQR: 2–5) (Mann–Whitney p < 0.001) (Table 2). Length of stay for RSV-positive cases among patients ≥ 5 years was not different from that for the negative-for-all-tested-viruses or only-positive-for-another-tested-respiratory-virus cases (Supplement Table S1). On the contrary, in the two age groups of children aged < 5 years, hospitalisation stay was longer for RSV-positive than for RSV-negative cases. For < 6-month-olds, the median stay for RSV positive patients  was 4 days (IQR: 3–6) vs a median of 3 days (IQR: 2–4) for RSV-negative patient (Mann–Whitney p < 0.001). For 6-month-to-4-year-olds, the median stay for RSV-positive patients was 4 days (IQR: 3–5) vs a median stay of 3 days for RSV-negative patients (IQR: 2–5; Mann–Whitney p = 0.055) (Supplement Table S1).

Twenty-two patients died during hospitalisation (four patients aged between 45 and 64 years, as well as 18 adults aged ≥ 65 years). All four patients aged between 45 and 64 years had more than one comorbidities. Among the patients ≥ 65 years, two had no report of comorbidity and five had only one comorbidities. By comparison, among the 35 patients aged between 45 and 64 years who survived, one had no comorbidity, 16 had one and 18 had more than one. Among the 110 surviving patients ≥ 65 years, one had no comorbidity, 40 had one and 69 had more than one. Overall, other kinds of complications than death were more common among children aged ≥ 5 years (97/182) than among those aged < 5 years (128/326; two-sided Fisher exact test p = 0.003). Among the latter, some complications seemed more common among the RSV-positive patients: pneumonia (25/33), requirement for respiratory assistance (82/103), ARDS (12/15) and transfer to ICU (6/6) (Table 2).

## Discussion

This pilot study showed that Belgian SARI network built to assess the severity of influenza viruses is able to capture the RSV activity if started earlier than usual. The beginning (week 41 2018) and peak (week 49 2018) of the RSV epidemic could be observed in this investigation and may have been missed by the usual SARI surveillance that started in week 2 2019 when the number of RSV positive samples had already considerably declined. The in-house RT-qPCRs allowed circulating RSVs to be typed and would allow to follow the change in type dominance if the current surveillance was extended to other years in the future [17,18]. The targeted number of patients to be included in the RSV surveillance per year [10] would have been reached if the whole network of six hospitals had taken part. This pilot study also showed that the Belgian SARI network could be used to evaluate the severity of RSV without much adaptation. The SARI network also has the advantage to operate based on a clear case definition and to allow the collection of data on several risk factors and severity indicators. 

It nonetheless appears that the two main populations of interest (children, especially those aged < 6 months, and older adults) present differences that need to be considered. Having a good case definition for the different age groups represents one of the challenges in the implementation of a surveillance system for RSV, as high sensitivity is required to avoid an underestimation of the RSV burden [19]. In this pilot study, we purposefully chose to employ the SARI case definition that has been used by the SARI network for several years, while only being less stringent for the fever criteria. This was deliberate in order to evaluate the information that could be readily obtained from our network, but this clearly represents a limitation of our study [20]. The data obtained during one season of surveillance were moreover not sufficient to perform robust measures of association between RSV and comorbidities or complications. In addition, how the comorbidities and the complications were defined and evaluated was left to each hospital to decide. Such lack of harmonisation also represents a limitation of the study that could be improved in the future. Another problem was the lack of information on the patients who did not consent to participate in the study. Of course, it would be impossible to obtain detailed information, but it could be useful to at least obtain the number of individuals lost to the surveillance and whether this should be taken into account in the analysis.

Nevertheless, the SARI case definition we used, even with a less strict fever criterion, allowed to follow RSV circulation in the children population aged < 5 years and to identify comorbidities/indicators potentially associated with disease severity. Wheezing, rhinitis and tachypnoea seem like notable additional clinical signs associated with RSV-positivity in children, as already described in a previous study [16], and would need to be included in a second pilot study to fully assess their relevance in increasing the case definition sensitivity. Extending the case definition to include other symptoms such as sepsis and apnoea might also need to be considered for children < 6 months, as recommended in WHO documents for RSV surveillance [10,14,15]. This would probably allow to better address the severity of disease caused by RSV in this age group, as these symptoms might be more frequently associated to severe forms [21,22]. Improving the case definition might be more challenging for older adults: the combination of clinical signs used here did not seem specific enough. Dyspnoea was more often reported in this age group, but irrespective of the RSV test result. However, a specific case definition might not be the most important point for a meaningful RSV surveillance. Assessment of the risk factors and RSV severity in this population would probably benefit more from a broader range of respiratory pathogens to be tested, including bacteria and fungi [23], in order to better evaluate the exact contribution of RSV.

These adaptations would have consequences on the actual SARI surveillance. To maintain a simple system, the case definition should not change during the whole surveillance period. The currently used SARI definition has proven effective to evaluate the severity of influenza viruses. Slightly extending it to better fit RSV would lead to an increase number of SARI cases being identified during the overall period of surveillance, which would then run from week 40 to week 20 of the following year. Since starting at week 40 would almost double the length of the surveillance, this could potentially result in almost doubling the number of collected samples. A broader case definition, with additional possible clinical signs and without fever, would lead to even more patients meeting the criteria and being enrolled. This would, however, not prevent us to identify the subset of cases responding to the more specific influenza case definition, and thus, it should not hamper us from evaluating the specific severity associated to influenza viruses. On the contrary, a broader case definition could allow the current surveillance of influenza viruses to become a surveillance system for several respiratory viruses of public health importance, such as RSV, human metapneumoviruses, and coronaviruses including the newly emerged severe acute respiratory syndrome coronavirus 2 (SARS-CoV-2) virus [3,24-27]. That being said, since the current SARI surveillance relies on the good will of the participating hospitals, the extra workload would require additional funding, as more personnel and more reagents would be required both at the hospital sites and at the national influenza centre. Given the benefits that would be gained in terms of better understanding the burden of respiratory viruses, such evolution of the SARI surveillance system might prove a more economic option on the long run.

To conclude, we showed that the implementation of a RSV surveillance based on our influenza SARI surveillance is feasible and would contribute to the objectives defined by WHO, but it would only be of interest if it can be sustained for several years. With SARI surveillance being more frequently implemented in several European countries, our study provides an example on how this surveillance can be used to gain information on RSV epidemiology.

## Supplementary Data

142000 Supplementary Material
