# Supplementary material for: Capturing respiratory syncytial virus season in Belgium using the influenza severe acute respiratory infection surveillance network, season 2018/19
Source: Euro Surveill. 2020 Oct 1;25(39):1900627. doi: 10.2807/1560-7917.ES.2020.25.39.1900627 (PMC7531071; doi:10.2807/1560-7917.ES.2020.25.39.1900627)
Supplement: Supplementary Material [file 1900627_Supplement.pdf]

Supplement Table S1

Complement to Table 1: detailed numbers of patients by age groups for symptoms, comorbidities, complications and hospitalization stay, Belgium, week 41-2018 to week 02-2019 (n=508).

This supplementary material is hosted by Eurosurveillance as supporting information alongside the article 'Capturing Respiratory Syncytial Virus season in Belgium using influenza Severe Acute Respiratory Infection surveillance network (season 2018-2019)' on behalf of the authors who remain responsible for the accuracy and appropriateness of the content. The same standards for ethics, copyright, attributions and permissions as for the article apply. Eurosurveillance is not responsible for the maintenance of any links of email addresses provided therein.

		RSV-negative				RSV-positive				Total	
		Negative for all		Positive for another[a]		RSV only		RSV co-infection[a]			
		n	%	n	%	n	%	n	%	n	%
overall		82		109		232		85		508	
age group	<5y	18[b]	22.0	55	50.5	173	74.6	80	94.1	326	64.2
	≥5y	64	78.0	54	49.5	59	25.4	5	5.9	182	35.8
symptom (yes)											
<6m	fever	6	100.0	23	95.8	109	93.2	40	95.2	178	94.2
	cough	6	100.0	21	87.5	114	97.4	37	88.1	178	94.2
	dyspnoea	3	50.0	7	29.2	45	38.5	16	38.1	71	37.6
6m-4y	fever	11	91.7	28	90.3	55	98.2	37	97.4	131	95.6
	cough	11	91.7	29	93.5	49	87.5	33	86.8	122	89.1
	dyspnoea	7	58.3	12	38.7	33	58.9	24	63.2	76	55.5
5-64y	fever	20	90.9	11	91.7	15	88.2	3	100.0	49	90.7
	cough	19	86.4	11	91.7	16	94.1	3	100.0	49	90.7
	dyspnoea	14	63.6	11	91.7	12	70.6	2	66.7	39	72.2
≥65y	fever	28	66.7	34	81.0	32	76.2	2	100.0	96	75.0
	cough	30	71.4	35	83.3	34	81.0	2	100.0	101	78.9
	dyspnea	36	85.7	36	85.7	36	85.7	2	100.0	110	85.9
comorbidity[c]											
<6m	none	6	100.0	20	83.3	105	89.7	35	83.3	165	87.3
	yes	0	0.0	4	16.7	12	10.3	7	16.7	24	12.7
	chr. respi.	0	-	0	-	3	-	2	-	5	-
	chr. cardio.	0	-	0	-	1	-	0	-	1	-
	neuromusc.	0	-	0	-	1	-	0	-	1	-
	premature	0	-	3	-	2	-	4	-	9	-
	tobacco	0	-	1	-	2	-	1	-	4	-
6m-4y	none	8	66.7	22	71.0	35	62.5	30	78.9	95	69.3
	yes	4	33.3	9	29.0	21	37.5	8	21.1	42	30.7
	chr. respi.	0	-	2	-	4	-	1	-	7	-
	asthma	1	-	1	-	2	-	2	-	6	-
	chr. cardio.	0	-	0	-	1	-	1	-	2	-
	renal insuf.	0	-	0	-	1	-	0	-	1	-
	immunodef.	0	-	1	-	3	-	1	-	5	-
	neuromusc.	0	-	1	-	5	-	0	-	6	-
	premature	0	-	2	-	4	-	3	-	9	-
	tobacco	0	-	0	-	1	-	2	-	3	-
5-64y	none	4	18.2	2	16.7	4	23.5	0	0.0	10	18.5
	yes	18	81.8	10	83.3	13	76.5	3	100.0	44	81.5
	chr. respi.	10	-	4	-	3	-	1	-	18	-
	asthma	2	-	1	-	3	-	1	-	7	-
	chr. cardio.	2	-	3	-	2	-	1	-	8	-
	renal insuf.	1	-	1	-	2	-	2	-	6	-
	hep. insuf.	1	-	2	-	2	-	0	-	5	-
	obesity	3	-	2	-	2	-	1	-	8	-
	diabetes	1	-	1	-	2	-	0	-	4	-
	immunodef.	3	-	2	-	6	-	1	-	12	-
	neuromusc.	2	-	3	-	0	-	0	-	5	-
	unknown	1	-	0	-	0	-	0	-	1	-
	tobacco	1	-	2	-	0	-	2	-	5	-

≥65y	none	2	4.8	1	2.4	0	0.0	0	0.0	3	2.3
	yes	40	95.2	41	97.6	42	100.0	2	100.0	125	97.7
	chr. respi.	17	-	16	-	18	-	2	-	53	-
	asthma	2	-	5	-	5	-	0	-	12	-
	chr. cardio.	16	-	21	-	17	-	0	-	54	-
	renal insuf.	10	-	12	-	12	-	1	-	35	-
	hep. insuf.	3	-	4	-	2	-	0	-	9	-
	obesity	10	-	3	-	2	-	0	-	15	-
	diabetes	7	-	11	-	6	-	2	-	26	-
	immunodef.	3	-	12	-	13	-	1	-	29	-
	neuromusc.	3	-	6	-	6	-	0	-	15	-
	unknown	1	-	0	-	0	-	0	-	1	-
	tobacco	0	-	1	-	2	-	0	-	3	-
antibiotics											
<6m	no	2	33.3	13	54.2	83	70.9	32	76.2	130	68.8
	yes	4	66.7	9	37.5	29	24.8	10	23.8	52	27.5
	unknown	0	-	0	-	0	-	0	-	0	-
	missing	0	-	2	-	5	-	0	-	7	-
6m-4y	no	7	58.3	17	54.8	26	46.4	21	55.3	71	51.8
	yes	5	41.7	13	41.9	29	51.8	17	44.7	64	46.7
	unknown	0	-	0	-	0	-	0	-	0	-
	missing	0	-	1	-	1	-	0	-	2	-
5-64y	no	5	22.7	1	8.3	2	11.8	2	66.7	10	18.5
	yes	16	72.7	10	83.3	14	82.4	1	33.3	41	75.9
	unknown	0	-	0	-	1	-	0	-	1	-
	missing	1	-	1	-	0	-	0	-	2	-
≥65y	no	12	28.6	5	11.9	9	21.4	0	0.0	26	20.3
	yes	28	66.7	37	88.1	31	73.8	2	100.0	98	76.6
	unknown	1	-	0	-	0	-	0	-	1	-
	missing	1	-	0	-	2	-	0	-	3	-
complication[d]											
<6m	no	5	83.3	22	91.7	73	62.4	26	61.9	126	66.7
	yes[e]	1	16.7	2	8.3	44	37.6	16	38.1	63	33.3
	pneumonia	0	-	1	-	5	-	2	-	8	-
	ICU	0	-	0	-	3	-	2	-	5	-
	ARDS	0	-	1	-	5	-	3	-	9	-
	resp. assis.[f]	1	-	1	-	40	-	14	-	56	-
6m-4y	no	4	33.3	15	48.4	31	55.4	22	57.9	72	52.6
	yes	8	66.7	16	51.6	25	44.6	16	42.1	65	47.4
	pneumonia	2	-	5	-	10	-	8	-	25	-
	ICU	0	-	0	-	0	-	1	-	1	-
	ARDS	0	-	2	-	2	-	2	-	6	-
	resp. assis.	6	-	13	-	20	-	8	-	47	-
5-64y	no	10	45.5	4	33.3	8	47.1	1	33.3	23	42.6
	yes	12	54.5	8	66.7	9	52.9	2	66.7	31	57.4
	pneumonia	7	-	4	-	7	-	0	-	18	-
	ICU	5	-	4	-	3	-	0	-	12	-
	ARDS	1	-	2	-	1	-	0	-	4	-
	resp. assis.	5	-	4	-	3	-	2	-	14	-
≥65y	no	26	61.9	21	50.0	14	33.3	1	50.0	62	48.4
	yes	16	38.1	21	50.0	28	66.7	1	50.0	66	51.6
	pneumonia	10	-	16	-	14	-	1	-	41	-
	ICU	10	-	7	-	15	-	1	-	33	-
	ARDS	4	-	5	-	6	-	0	-	15	-
	resp. assis.	7	-	6	-	14	-	1	-	28	-
stay in hospital[g]											
<6m	median	3	-	3	-	4	-	4	-		
	min	2	-	1	-	1	-	1	-		
	25% perc.	2	-	2	-	3	-	2	-		
	75% perc.	5.5	-	3.8	-	6	-	5.3	-		
	max	7	-	9	-	16	-	14	-		
6m-4y	median	3.5	-	3	-	4	-	4	-		
	min	2	-	1	-	1	-	1	-		
	25% perc.	2	-	2	-	3	-	3	-		

	75% perc.	5	-	4	-	5	-	5	-
	max	12	-	19	-	32	-	13	-
5-64y	median	7.5	-	10.5	-	10	-	8	-
	min	2	-	2	-	2	-	5	-
	25% perc.	3.8	-	7.3	-	4	-	5	-
	75% perc.	13.3	-	15.8	-	17.5	-	9	-
	max	31	-	104	-	49	-	9	-
≥65y	median	9	-	9.5	-	8.5	-	7.5	-
	min	1	-	2	-	1	-	7	-
	25% perc.	4.8	-	6	-	5	-	7	-
	75% perc.	14.3	-	16	-	14.3	-	8	-
	max	37	-	87	-	56	-	8	-

[a] other respiratory viruses tested: influenza virus types A and B, human metapneumoviruses, parainfluenzaviruses (types 1, 2, 3 and 4), coronaviruses (CoV-OC43, CoV-NL63 and CoV-229E), adenoviruses, picornaviruses of the *rhinovirus* and *enterovirus* genera, specific enterovirus D68, parechovirus, and bocavirus

[b] number of patients and percentage within laboratory result category

[c] chr. respi.: chronic respiratory disease; chr. cardio.: chronic cardiovascular disease; renal insuf.: renal insufficiency; hep. insuf.: hepatic insufficiency; immunodef: immunodeficiency; neuromusc.: neuromuscular disease

[d] other than death

[e] at least one of the following: detection of pneumonia based on chest radiography; admission in intensive care unit (ICU); development of acute respiratory distress syndrome (ARDS); requirement for respiratory assistance (resp. assis.)

[f] including invasive and non-invasive respiratory assistance

[g] in days; perc.: percentile
